# Effect of the Interactions between Oppositely Charged Cellulose Nanocrystals (CNCs) and Chitin Nanocrystals (ChNCs) on the Enhanced Stability of Soybean Oil-in-Water Emulsions

**DOI:** 10.3390/ma15196673

**Published:** 2022-09-26

**Authors:** Sanjiv Parajuli, Mohammad Jahid Hasan, Esteban E. Ureña-Benavides

**Affiliations:** Department of Biomedical Engineering and Chemical Engineering, The University of Texas at San Antonio, San Antonio, TX 78249, USA

**Keywords:** chitin nanocrystals (ChNCs), cellulose nanocrystals (CNCs), interaction, food-grade emulsions, emulsion stability

## Abstract

Chitin nanocrystals (ChNCs) and cellulose nanocrystals (CNCs) have been recently used to stabilize emulsions; however, they generally require significant amounts of salt, limiting their applicability in food products. In this study, we developed nanoconjugates by mixing positively charged ChNCs and negatively charged CNCs at various ChNC:CNC mass ratios (2:1, 1:1, and 1:2), and utilized them in stabilizing soybean oil–water Pickering emulsions with minimal use of NaCl salt (20 mM) and nanoparticle (NP) concentrations below 1 wt%. The nanoconjugates stabilized the emulsions better than individual CNC or ChNC in terms of a reduced drop growth and less creaming. Oppositely charged CNC and ChNC neutralized each other when their mass ratio was 1:1, leading to significant flocculation in the absence of salt at pH 6. Raman spectroscopy provided evidence for electrostatic interactions between the ChNCs and CNCs, and generated maps suggesting an assembly of ChNC bundles of micron-scale lengths intercalated by similar-size areas predominantly composed of CNC. The previous measurements, in combination with contact angles on nanoparticle films, suggested that the conjugates preferentially exposed the hydrophobic crystalline planes of CNCs and ChNCs at a 1:1 mass ratio, which was also the best ratio at stabilizing soybean oil–water Pickering emulsions.

## 1. Introduction

Emulsions, thermodynamically unstable heterogeneous mixtures of immiscible liquids, are a form of advanced materials that require addition of a stabilizer to prevent a phase separation induced by the fusion of droplets during storage. Surfactants and nano- or micro-particles are commonly used as stabilizers to prepare these emulsions as they stabilize the liquid/liquid interface. Although both surfactants and nanoparticles (NPs) adsorb to the interface, the mechanisms by which they stabilize emulsions vary significantly; however, adsorption to the interface can be considered a prerequisite for stabilization [1,2,3,4]. In general, emulsions prepared using NPs are more stable than surfactant-stabilized emulsions due to the high energy of adsorption and large interfacial elasticity associated with NPs [4,5,6]. Both synthetic and biobased NPs are used to stabilize emulsions, but those of natural origin may be preferred for pharmaceutical, biomedical, and food applications as they often offer enhanced biocompatibility.

Cellulose and chitin are both abundant biopolymers, which have gained interest in the last few decades to develop advanced materials due to their unique physical and chemical properties along with their associated low cost, and low toxicity to humans and the environment [4,7,8,9]. Chemically, cellulose is a carbohydrate polymer of β-D glucopyranose, while chitin can be considered a derivatized cellulose; more specifically, it is a polymer of N-acetyl-D-glucosamine units. Both are linear polymer chains linked together by β (1–4) glycosidic bonds. In the case of cellulose nanocrystals (CNCs), their isolation using sulfuric acid introduces sulfonate groups to replace approximately 30% of the superficial primary hydroxyl groups in the C6 position, rendering them negatively charged over a broad range of pH [10,11,12]. Chitin nanocrystals (ChNCs), on the other hand, have acetamide groups on the C2 position of the D-glucopyranose unit, imparting a higher hydrophobicity compared to CNCs. During their isolation reaction, chitin fibers are partially deacetylated, resulting in exposed primary amino groups on their surface [13,14]. The surface charge on ChNCs, thus, depends on the pH of the medium, and they are protonated at pHs lower than their isoelectric point.

Chitin-based NPs have been extensively studied as Pickering emulsion stabilizers for food applications [13,14,15,16,17]. Cellulose nanocrystals have also been used in food-grade emulsions at varying degrees [18,19,20,21,22]. However, charged CNCs alone do not stabilize oil/water emulsions well unless an electrolyte is added to increase the stability of the emulsions [20]. In the case of ChNCs, the addition of salts also improves the stability of oil/water emulsions [14]. On the other hand, studies on Pickering emulsions have shown that emulsions prepared with mixtures of oppositely charged nanoparticles either do not require an electrolyte or required lower concentrations, compared to the emulsions prepared with a single type of nanoparticle [23,24,25]. It was found that oppositely charged latex and Ludox CL NPs formed nano-complexes that were able to stabilize oil–water emulsions without using any electrolytes [25]. Furthermore, Nallamilli et al. reported the stability of oil–water emulsions using two oppositely charged latex particles and discovered that at a particle number ratio of 1:1, the emulsions were most stable, while the positively or negatively charged particles alone were not able to stabilize any emulsions [24]. In another study, Li et al. synthesized Chitosan (CS)-functionalized cellulose nanocrystals (CNC/CS) and used them to stabilize oil–water emulsions and found that the CNC/CS stabilized emulsions had high resistance to temperature, pH, and salt [26].

Huan et al. studied the oil/water emulsion stability by combining positively charged nanochitin (NCh) and negatively charged cellulose nanofibers (CNF) to prepare stable emulsion systems [23] and found that CNF/NCh complexes greatly extend the range of emulsion stability conditions, favoring highly stable, green Pickering emulsions that are resistant to both creaming and coalescence. Similarly, Lv et al. showed in a recent work that oil/water Pickering emulsions could be made utilizing either cellulose or chitin nanofibrils, as well as their physical combinations [27]. In their study, they focused on the applicability of chitin and cellulose nanofiber mixtures in Pickering emulsions, while putting a lesser emphasis on particle–particle physical interactions.

There are few reports focusing on the combination of CNC and ChNC to produce stable emulsions. Though oppositely charged chitin and cellulose nanofibers (CNF) have been reported in two studies [23,27], the mixtures of shorter nanocrystals have not been explored much. There are several fundamental differences between CNFs and CNCs; the former are a few micrometers long, retain some amorphous regions, and are more flexible, whereas the latter are rigid, highly crystalline, and have lengths between 100 and 200 nm. In addition, the CNCs have been etched by acid hydrolysis, exposing a hydrophobic (200) crystalline plane, thus imparting a Janus-like character to the nanoparticle [28,29,30]. Analogous differences apply for the ChNCs used in this study compared to their nanofibrils. Moreover, in this study, nanoconjugates of CNC and ChNC at various ratios were developed and the fundamental interfacial and molecular interactions, as well as the structure of the nanoparticle conjugates, were studied by carefully characterizing the CNC/ChNC mixtures. The CNC/ChNC mixtures were then utilized to prepare stable food-grade soybean oil/water emulsions with low NPs and NaCl concentrations.

## 2. Materials and Methods

### 2.1. Materials

Commercially available CNCs were purchased from CelluForce Inc. (Montreal, QC, Canada) as a 6 wt% aqueous dispersion (NCV100-NAL90). The manufacturer synthesizes CNCs from the wood pulp by sulfuric acid hydrolysis. Chitin powder (shrimp shell), hydrochloric acid, sodium hydroxide (10 N), soybean oil (>99.5 purity), and n-heptane (>99.9% purity) were purchased from Fisher Scientific (Hampton, NH, USA) and were used as received.

### 2.2. Synthesis of Chitin Nanocrystals

Chitin nanocrystals (ChNC) were synthesized using procedures described elsewhere [31,32]. Briefly, chitin powder was added to 0.25 M HCl (1 g solid: 40 mL HCl) and stirred under ambient conditions for 30 min to demineralize chitin powder. The obtained chitin was filtered using Whatman No. 1 filter paper and rinsed with DI water. The cake obtained from the filtration was then subjected to mixing with 1.25 M NaOH at 70 °C (15 mL of NaOH per g of cake) for 24 h to remove proteins from chitin. The mixture was then washed multiple times with DI water and filtered. The purified chitin was then washed with acetone and ethanol to remove organic soluble compounds followed by rinsing with DI water. The chitin was then hydrolyzed with 3 M HCl (30 mL of HCl per g of chitin) at 90 °C under vigorous stirring for 90 min. The reaction was quenched using ice-cold water (10:1 volumetric ratio) and the reaction mixture was centrifuged to remove excess acid. The chitin nanocrystals (ChNC) thus produced were collected, suspended in DI water, and dialyzed against DI water until the pH of the surrounding media was constant. The ChNC suspension was then sonicated (5000 J/g-solid) and stored at 4 °C until further use.

### 2.3. Size Distributions of CNC and ChNC

Atomic force microscopy (AFM) in tapping mode (300 MHz, noncoated silicon nitride tip) was used to obtain the topographic images of CNC and ChNC using a Bruker Di Innova microscope from Bruker (Billerica, MA, USA). A drop of diluted CNC or ChNC suspension (0.0002 wt%) was deposited onto a freshly cleaved mica surface (V1 AFM Mica Discs, 10 mm diameter, Ted Pella Inc., Redding, CA, USA) and dried overnight in a desiccator. The images were processed using Gwyddion software to measure the dimensions of both NPs. A minimum of 175 rod-shaped individual nanocrystals were measured to obtain the size distributions.

### 2.4. Surface Charge of CNC and ChNC

Conductometric titrations were completed to estimate the charge on ChNC and CNC surfaces. In the case of CNCs, the nanoparticles were first dialyzed against hydrochloric acid (1.0 M) to remove sodium counterions and protonate the superficial sulfate groups. The surrounding medium was replaced daily until constant pH. The protonated CNCs were then dialyzed against DI water to remove excess acid, following the same method. The resulting CNC dispersion had a pH of 3.4; then, 50 mL of 0.2 wt% CNC was titrated with 0.05 M NaOH while measuring the pH and electrical conductivity [5,33,34]. In the case of ChNCs, a 5.85 mL aliquot of 1.71 wt% dispersion was diluted with 1 mM HCl to a final volume of 100 mL and a concentration of 0.1 wt%. The sample was then titrated with 0.02 M NaOH [13,14,35].

### 2.5. Zeta Potential and Aggregate Sizes

The ζ-potential and mean hydrodynamic diameter of ChNC and CNC were measured as a function of pH using a Malvern ZetaSizer Nano ZS (Westborough, MA, USA) for diameters lower than 300 nm. Larger aggregate sizes were measured using a laser diffraction particle size analyzer, Horiba Partica LA-960V2 (Irvine, CA, USA). The ζ-potential and size of ChNC-CNC nanoconjugates were also measured for ChNC:CNC mass ratios of 3:0, 2:1, 1:1, 1:2, and 0:3, where 3:0 represents pure chitin and 0:3 represents pure CNC, while keeping the total NP concentration and the pH constant at 0.2 wt% and 6.0, respectively.

### 2.6. X-ray Diffraction

Powder X-ray diffractograms (XRD) were collected using Malvern Panalytical Empyrean Nano Edition (Westborough, MA, USA). Individual ChNC and CNC along with their mixtures were freeze-dried, loaded in a sample holder, and exposed to X-ray scans from 2θ angles of 5° to 60° at a step time of 0.6 s/step (0.003°/step) and sample revolution of 4 rev/min at 25 °C with Cu Kα radiation (λ = 0.1540 nm).

### 2.7. Raman Spectroscopy and Mapping

Raman spectra and images were obtained using Horiba LabRam HR-Evolution (Piscataway, NJ, USA) equipped with 600 grooves/mm grating, 532 nm laser excitation, CCD camera detection, and an MPlan N 100×/0.9 objective from Olympus (Waltham, MA, USA). Raman maps were obtained with a 10 nm step size. The spectra were collected using an acquisition time of 1 s and 500 accumulations to improve the signal-to-noise ratio. Individual ChNC, CNC, and ChNC-CNC conjugates dispersions in DI water were drop-casted on a freshly cleaved mica surface and dried under ambient conditions prior to obtaining their Raman spectra. The spectra of different ChNC-CNC nanoconjugates were examined for spectral signatures to examine probable interactions between ChNC and CNC.

### 2.8. Interfacial Tension and Wettability

The equilibrium interfacial tension (γ_ow_) between soybean oil and aqueous phases containing ChNC, CNC, and ChNC-CNC nanoconjugates was measured under ambient conditions using a Kruss Scientific DSA100S (Matthews, NC, USA) optical tensiometer as a function of pH and NP mass ratio. A pendant droplet of a 20 mM NaCl solution was formed on the tip of the needle submerged in soybean oil, while measuring γ_ow_ for 60 min. Contact angles were measured using the sessile drop method to describe the macroscopic wettability of NP films (drop-casted and air-dried) submerged in soybean oil. The static equilibrium contact angle was measured after allowing the sessile drop of a 20 mM NaCl solution to equilibrate on NP films for 200 s.

### 2.9. Emulsions Stability

A series of preliminary emulsion stability screening experiments were carried out to determine the salt and nanoparticle concentrations needed for this study. Several emulsions were prepared with NaCl concentrations ranging from 0 to 100 mM and NP concentrations ranging from 0.05 to 1.0 wt%. The lowest concentrations that resulted in a low emulsion stability for CNC and ChNC alone, but were stable for mixed NP dispersions, were chosen for the remaining experiments, as indicated in the Supplementary Materials (SM) Figures S1–S4. All emulsions were prepared using 20 wt% soybean oil as stable emulsions are expected at this volume fraction [5,36]. After screening, all aqueous phases were prepared using 20 mM NaCl and a total NP concentration of 0.2 wt%. The pH of the aqueous suspensions was adjusted to 6 prior to making the emulsions, as determined by the zeta potential measurements. Emulsion stability was studied in terms of macroscopic creaming between times of 0 h and 24 h for screening experiments, and over a period of 4 weeks for long-term stability experiments. In addition, corresponding microscopy pictures of the emulsion droplets were recorded with an optical microscope and the size distribution of emulsion droplets was measured using a Horiba LA-960V2 (Kyoto, Japan). Finally, the NP orientation on the surface of the emulsion droplets was studied using SEM images of stable emulsion droplets taken using an FE-SEM, Hitachi, S-5500 (Tokyo, Japan) after the emulsions were dried with an Automated Critical Point Dryer (Leica EM CPD300, Leica Microsystems GmbH, Wetzlar, Germany) using the following program: slow CO_2_ admittance with 120 s delay, 16 exchange cycles, followed by a slow heating to 40 °C and slow gas discharge. Before drying in the automated CPD, the emulsions were diluted to 1-part emulsion per 10-part acetone and were fixed in an SEM substrate to dry overnight and then were transferred to a meshed container to dry using CPD. For the SEM images, soybean oil was replaced with n-Heptane due to its lower boiling point.

## 3. Results and Discussion

### 3.1. Characterization of Individual Cellulose and Chitin Nanocrystals

Cellulose and chitin nanoparticles were studied using AFM to elucidate their morphological properties. Topographic AFM images revealed that both CNC and ChNC were anisotropic rod-shaped particles with average lengths of 130 ± 59 and 193 ± 60 nm, respectively, as shown in Figure S5. The particle charge was determined by conductometric titration. Chitin had a degree of deacetylation of 13.0%, equivalent to a charge of 4.11 × 10^20^ e^−^/g ChNC, while for CNC, the sulfur content was 0.435%, or 8.45 × 10^19^ e^−^/g ChNC (Figure S6). These charges, however, are only accurate when all amine groups are fully protonated in the case of chitin, or when all sulfate groups are fully deprotonated for CNC. As the pH of the dispersion changes, so does the net particle charge, as described in the following section.

### 3.2. Colloidal Stability of ChNC-CNC Nanoconjugates

**Figure 1:**
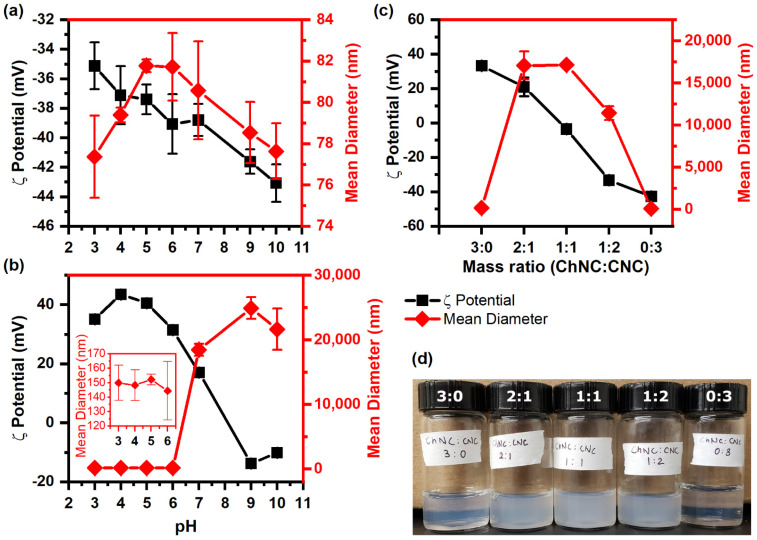
ζ-potential and mean aggregate size of (**a**) CNC, (**b**) ChNC, and (**c**) ChNC-CNC nanoconjugates at pH 6. (**d**) Picture of ChNC-CNC complexes in aqueous dispersion. In (**a**), the CNCs show a negative charge over the entire pH range with stable hydrodynamic diameters between 76 and 82 nm, indicating no aggregation. In (**b**), the ChNCs are positively charged below the isoelectric point at pH 8 and negatively charged above it, while their hydrodynamic diameter indicates no aggregation at pH 6 and below, but significant aggregation at pH 7 and above. In (**c**), the combination of ChNCs and CNCs neutralize the charges, leading to significant flocculation in the mixed samples.

The colloidal stability of individual nanoparticles and nanoconjugates in DI water was examined in terms of ζ-potential and average aggregate size. Figure 1a,b show the ζ-potential and average aggregate size of CNCs and ChNCs, respectively, from pH 3 to 10. The CNCs remained negatively charged for the entire pH range by virtue of the highly acidic sulfate groups on their surface, which remained mostly deprotonated over the range of conditions tested [34,37,38]. The ζ-potential of CNCs, however, became more negative as the pH increased, from −35 ± 2 mV at pH 3 to −43 ± 1 mV at pH 10, presumably due to the adsorption of hydroxide ions on their surface. As a consequence of the highly negative charge, the hydrodynamic diameter of CNCs (reported as mean diameter) remained approximately constant around 80 nm. In the case of ChNCs (Figure 1b), their primary amine groups (resulting from partial deacetylation of chitin) became protonated at acidic pHs; thus, the ζ-potential remained positive from pH 3 to 7, with a maximum of 43.5 ± 0.3 mV at pH 4 and an isoelectric point close to 8. The hydrodynamic diameter remained approximately constant near 149 nm from pH 3 to 6, when the ζ-potential was higher than 30 mV. Increasing the pH to 7 (ζ = 17.1 ± 0.8 mV) and above induced a drastic aggregation of the ChNC, resulting in volume-average diameters (reported as mean diameter) ranging from 18.5 ± 9 to 25 ± 2 µm. Based on these results, pH 6 was chosen for the remainder of the paper to prepare ChNC and CNC mixtures; at this pH, the ChNCs and CNCs were colloidally stable on their own, but had opposite charges of similar magnitude with ζ-potentials of 31.5 ± 0.2 mV and −39 ± 2 mV, respectively. It is expected that charge neutralization would occur when mixing both nanomaterials in similar proportions at this and lower pHs.

The ζ-potential of ChNC-CNC nanoconjugates is shown in Figure 1c; as expected, it decreased with an increasing mass fraction of CNC, while at a mass fraction of 1:1, the ζ-potential was close to zero (−4 ± 2 mV). The positively charged ChNC and negative CNC are likely to interact with each other primarily via electrostatic interactions. When they are mixed, the amine groups on the ChNC surface are attracted to the sulfate groups on the CNCs, leading to particle charge neutralization and aggregation. In fact, the mean diameter of the nanoconjugates increased rapidly to 17 ± 2 µm for a 2:1 ChNC:CNC ratio (ζ-potential = 21 ± 5 mV), and then started to decrease beyond a mass ratio of 1:2.

Nanoconjugates with a ChNC:CNC mass fraction of 1:2 had a large negative ζ-potential (−33.3 ± 1 mV). At high ζ-potential values (>±30 mV), one would expect a relatively small particle size; however, as evidenced by the cloudiness of the dispersion in Figure 1d, and the measured aggregate size of 11.4 ± 0.8 µm, it is clear that these nanoconjugates were aggregated. The results suggest that attractive electrostatic forces remained highly dominant for this mixed sample, keeping the oppositely charged ChNCs and CNCs in close proximity inside aggregated structures that possibly had a higher local concentration of negatively charged CNCs on their outer regions, resulting in a net negative charge.

### 3.3. Molecular Interactions and Structures of ChNC and CNC

**Figure 2:**
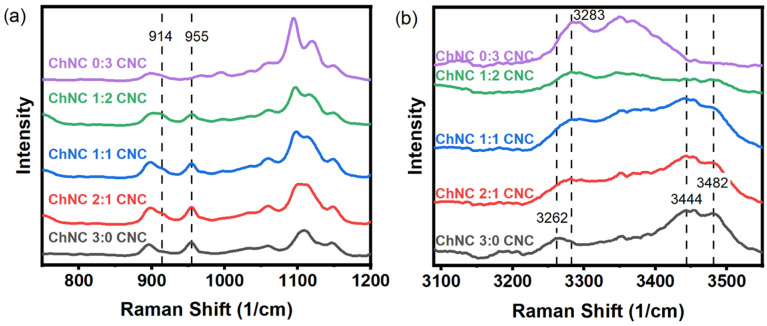
Raman spectra of ChNC, CNC, and ChNC-CNC nanoconjugates from (**a**) 750 to 1200 cm^−1^, and (**b**) 3100 to 3550 cm^−1^. In (**a**), evidence of a sulfate-ammonium interaction is observed by the shoulder at 914 cm^−1^ and the peak at 955 cm^−1^. Panel (**b**) shows an alteration of the hydrogen bond pattern as the ChNCs and CNCs are mixed together in addition to the presence of -NH stretching bands at 3444 and 3482 cm^−1^ for chitin that are not present in CNC.

Molecular interactions between ChNC and CNC in nanoconjugates and by themselves were studied using Raman spectroscopy. Under the study conditions, ChNC and CNC surfaces were oppositely charged; thus, electrostatic forces between these nanoparticles were likely to be the most dominant intermolecular force. Evidence for such an interaction was observed by a weak shoulder at 914 cm^−1^ and a peak at 955 cm^−1^ (Figure 2a). Such vibrations were assigned to the O-S-O groups interacting with the protonated amine groups on the ChNC surface. Dhumal et al. studied the Raman spectrum of the ionic liquid 1-ethyl-3-methylimidazolium ethyl sulfate and assigned two peaks at 915 and 958 cm^−1^ to O-S-O symmetric stretching, corresponding to the oxygen atoms directly interacting with the imidazolium cation [39,40]. Chitin shows a C-N stretching vibration at 954 cm^−1^ [41], which overlaps with the 955 cm^−1^ vibration from the sulfate groups. If the peak at 955 cm^−1^ in Figure 2a was only due to the C-N stretching vibration, its size would diminish as the chitin content is reduced; instead, its intensity remained approximately constant for samples with lower chitin and higher cellulose content, while disappearing completely for samples with CNCs only. Such a behavior suggests that both vibrations are effectively overlapped, yet they are not present when the CNCs do not interact with ChNCs. On the other hand, the shoulder at 915 cm^−1^ was only present for the samples containing mixed CNCs and ChNCs, suggesting that it arose from the interaction between both nanoparticles. Stretching vibrations from -OH and -NH bonds were observed in the 3250 to 3550 cm^−1^ region (Figure 2b). In the case of CNCs, a broad peak at 3283 cm^−1^ was assigned to -OH stretching in positions C2 and C6, which are hydrogen-bonded in consecutive anhydroglucose rings, while the peak at 3351 cm^−1^ was likely due to vibrations arising from the oxygens in positions C2, C6, C3, and C5, all of which interact through intra- and inter- chain hydrogen bonds [42]. For chitin, an acetyl amine group replaces the C2 hydroxyl group, resulting in an acetylated glucosamine ring. This substitution resulted in significant changes in the hydrogen bond pattern and the appearance of -NH stretching vibrations. Thus, a smaller broad band was observed at 3262 cm^−1^, while two larger broad bands showed up at 3444 and 3482 cm^−1^. These characteristic chitin and cellulose vibrations appeared to vary according to their concentration as the ChNC-to-CNC ratio was modified. An estimated 13% of the acetyl amine groups in the ChNC were deacetylated and protonated. These should result in -NH_3_^+^ stretching vibrations between 3000 and 3100 cm^−1^ [43]; however, they were not readily observed, likely due to their small concentration. Full spectra from 200 to 3600 cm^−1^ are provided in Supplementary Materials Figure S7.

**Figure 3:**
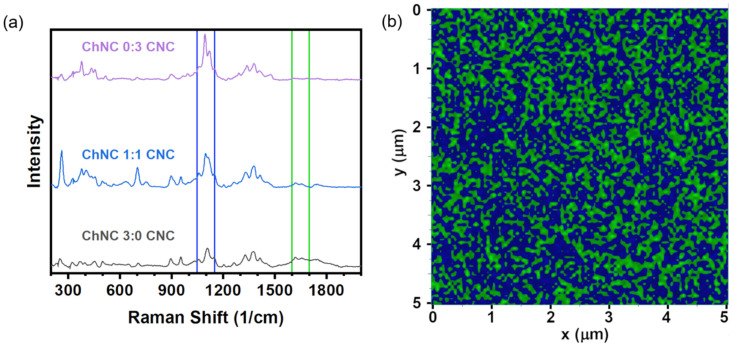
(**a**) Raman spectra of pure CNC, 1:1 ChNC:CNC, and pure ChNC films showing the bands used for Raman mapping. (**b**) Raman signal map of a 1:1 ChNC/CNC thin film, providing an approximate distribution of ChNC and CNC on the film. The colors represent vibrations within 1050–1150 cm^−1^ (blue) and 1600–1700 cm^−1^ (green).

A Raman signal map is shown in Figure 3 representing a distribution of CNC and ChNC characteristic vibrations on a thin ChNC/CNC thin film. Despite having used a scan step size of 10 nm, the radius of the laser beam was estimated to be $1.22\lambda /\left(2NA\right)=361\;\mathrm{nm}$, where λ is the laser wavelength and NA is the objective numerical aperture. Thus, each positional data point in the obtained maps had somewhat convoluted information. To reduce beam size errors, each pixel color depends on the highest-intensity Raman band at any given position; therefore, they approximately represent the location of CNC and ChNC on the film. The maps were built from Raman shifts at 1600–1700 cm^−1^ (green regions) corresponding to carbonyl vibration bands in ChNC, and 1050–1150 cm^−1^ (blue regions) from skeletal bending and stretching, as well as glycosidic O-C-O bond vibrations [42]. The green region is exclusive of the ChNC, while the shifts within the blue region are signals present in both CNC and ChNC; however, the vibrations were much stronger in the CNC. Thus, the green regions represent chitin-only parts of the film, while the blue regions are predominantly (or exclusively) CNC. The map revealed a uniform distribution of CNC and ChNC signals over the thin film with elongated clusters of ChNC of micron-scale lengths. An accurate quantification of the clusters’ widths could not be obtained, due to the relatively large beam diameter compared to their size.

**Figure 4:**
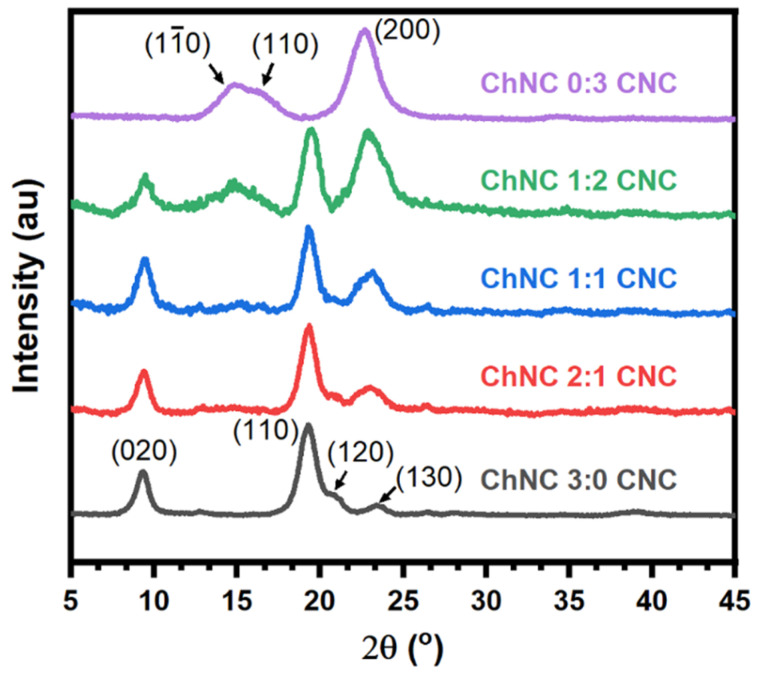
X-ray diffractograms of individual chitin (ChNC) and cellulose (CNC) nanoparticles along with ChNC-CNC nanoconjugates, showing a reduction in peak sizes as the concentration of nanoparticles is reduced.

Figure 4 shows X ray diffractograms of individual ChNC and CNC along with the nanoconjugates. For CNC, four main peaks were observed at 14.8°, 16.5°, and 22.7°, corresponding to the (1$\bar{1}$0), (110), and (200) crystalline planes of the monoclinic cellulose Iβ allomorph [44,45]. Interestingly, the (1$\bar{1}$0) and (110) reflections of CNC were significantly overlapped, presumably due to peak broadening arising from the small crystallite size, which was estimated to be about 6 nm. In the case of ChNC, the main reflections occurred at 9.3°, 19.3°, 20.8°, and 23.4°, arising from the (020), (110), (1,2,0), and (1,3,0) crystalline planes of the orthorhombic α-chitin [46,47,48,49]. X-ray diffractograms of ChNC-CNC nanoconjugates showed that all peaks were conserved, suggesting that the mixing of these nanoparticles did not affect their crystalline structure. As expected, the area of the peaks dropped as the concentration of each nanoparticle decreased for mixed samples.

### 3.4. Surface Properties of ChNC-CNC Nanoconjugates

**Figure 5:**
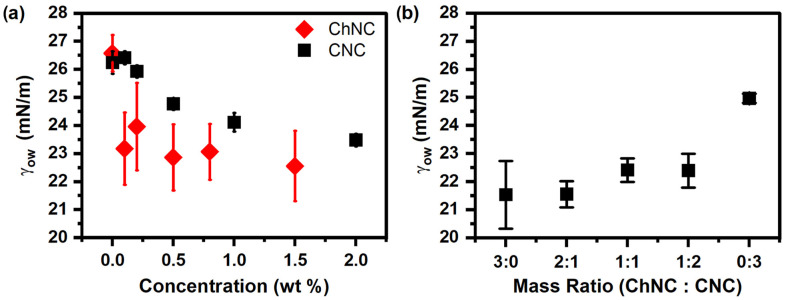
Interfacial tension (γ_ow_) between soybean oil and 20 mM NaCl solutions as a function of (**a**) CNC and ChNC concentration and (**b**) ChNC:CNC mass ratio. Both ChNC and CNC lower γ_ow_ by 4 and 3 mN/m, respectively, while addition of CNC to ChNC increases the γ_ow_.

The surface properties of CNC, ChNC, and ChNC-CNC nanoconjugates were studied in terms of the γ_ow_ between soybean oil and a 20 mM NaCl solution with dispersed nanocrystals under ambient conditions. Additionally, the three-phase contact angles (θ) of 20 mM NaCl solutions were measured on NP films submerged in soybean oil. Figure 5a shows the effect of nanocrystal concentration on interfacial tension for both ChNC and CNC. Increasing nanoparticle concentration decreased γ_ow_ between soybean oil and the aqueous phase, suggesting that both nanoparticles used in the study showed surface-active properties. It should be noted, however, that while there was a decrease in γ_ow_ between the oil and aqueous phases with increasing NP concentration, the reduction was minimal (~3 mN/m in the case of CNC and ~4 mN/m for ChNC), similar to other systems containing unmodified nanocelluloses, as described in the recent literature [4]. Figure 5a showed that addition of ChNC beyond 0.1 wt% did not significantly lower the γ_ow_, whereas increasing the CNC concentration up to 2 wt% resulted in a monotonous reduction. The more significant γ_ow_ reduction for ChNCs at low concentration was likely caused by their higher hydrophobicity (compared to CNC) arising from their superficial acetamido groups [13,14].

The effect of different mass ratios of nanoparticles on the γ_ow_ was also measured at a constant total NP concentration of 0.2 wt% for different ChNC-CNC nanoconjugates (Figure 5b). Increasing the mass fraction of CNC in a nanoparticle suspension appeared to slightly increase the interfacial tension between soybean oil and the nanoparticle suspensions. The result was expected as at 0.2 wt%, ChNCs lowered γ_ow_ by about 3 mN/m, while at the same concentration, CNCs had no significant effect. It is interesting to note that with the exception of the 0:3 samples (CNC only), all mass ratios resulted in approximately equal γ_ow_—within the error of the measurement—indicating that the conjugates retained the enhanced hydrophobicity of the ChNCs. Nevertheless, the effect of nanoconjugate addition on γ_ow_ was too small to have a significant effect on emulsion stability.

**Figure 6:**
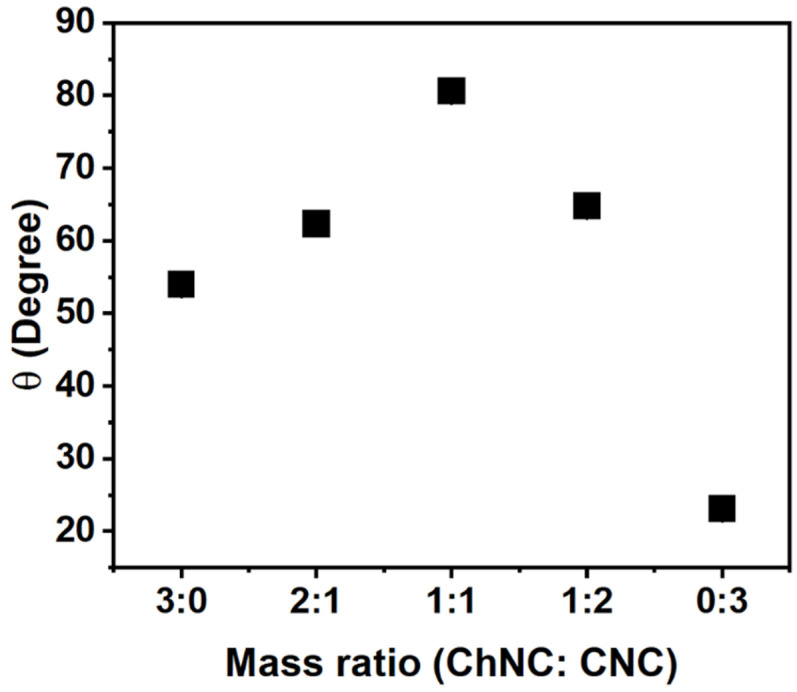
Contact angle of a 20 mM NaCl solution on nanocrystal films submerged in soybean oil as a function of ChNC:CNC mass ratio, indicating a maximum contact angle at a 1:1 mass ratio. The result provides evidence of a preferential exposure of hydrophobic planes when both nanoparticles are combined. The error bars are smaller than the markers; the standard deviations are, from left to right: 0.65°, 0.18°, 0.79°, 0.34°, and 0.43°.

A different stabilization mechanism is expected if the NP adsorption energy is high, which is typically evidenced by contact angles close to 90° [1,4,5,50]. In addition to interfacial tension measurements, contact angles were measured for 20 mM NaCl solutions on NP films prepared using different mass fractions of CNC and ChNC (0.2 wt% total). Figure 6 shows that the contact angles increased from 54.0 ± 0.7° to 80.6 ± 0.8° as the mass ratio of the nanoconjugates changed from 3:0 to 1:1 (ChNC:CNC). Upon further addition of CNC (1:2 and 3:0), the contact angle decreased to 23.1 ± 0.4° for films containing only CNC. The presence of a maximum contact angle at a 1:1 ratio was consistent with the zeta potential measurements (Figure 1c), which showed a ζ-potential close to zero for the same nanoconjugate composition. Such an observation is also consistent with prior reports that at low zeta potential, there is a rapid decline in water flux [51], and under such conditions, nanoconjugates are likely to have limited interaction with water, thus increasing their water contact angle. From a structural perspective, it is well known that the sulfate and hydroxyl groups on the CNC are exposed on the (110) and (1$\bar{1}$0) crystalline planes [28,29,30], while the ammonium and hydroxyl groups on the ChNC surface are mainly exposed on the (020) surface, as reported by Ogawa et al. and Silorski et al. [48,52]. If those hydrophilic surfaces are strongly attracted to each other by electrostatic and hydrogen bond forces, as evidenced by the ζ-potential and Raman studies, then it is likely that the more hydrophobic planes of each crystal, which mostly contain -CH groups, are exposed in the case of the nanoconjugates, giving rise to a higher contact angle. These hydrophobic surfaces are the (200) planes of the CNCs, and (120) and (1$\bar{2}$0) in the case of ChNCs [28,29,30,48,52]. Future work by the authors will attempt to quantify the orientation of the crystalline planes to confirm such a morphology.

The contact angle measurements in Figure 6 suggest a preferred aggregation of both types of nanocrystals through the more hydrophilic surfaces. It is, thus, expected that ChNC-CNC nanoconjugates with a mass ratio of 1:1 would be conducive to more stable soybean oil/water emulsions. It has been established in extensive prior research on Pickering emulsions that contact angles closer to 90° provide surface properties favorable for particle adsorption at the fluid–fluid interface and, thus, an improved ability to stabilize emulsions and foams [34,50,53,54].

### 3.5. Emulsion Stability

Cellulose and chitin nanocrystals were used to stabilize emulsions containing 20 wt% soybean oil. A preliminary screening of emulsion creaming over 24 h is shown in the Supplementary Materials document to determine the most appropriate salt concentration within 0 to 100 mM (Figure S1), NP concentration from 0.05 to 1 wt% (Figure S2), and NP mass ratio (Figure S3). In addition, a 4-week study of emulsion creaming was performed at 0.02 M NaCl, a 1:1 ChNC:CNC mass ratio, and total nanoconjugate concentrations ranging from 0.1 to 0.8 wt% (Figure S4). The preliminary screening measurements in Figures S2 and S3 indicated that ChNCs were more effective in producing stable emulsions, compared to CNC under identical conditions. The experiments also corroborated expected trends established in the literature, such as an increased stability at higher salt concentrations and at higher NP content [30,55,56]. However, deviations from such a behavior have been observed in systems at ionic strengths of 0.65 M and above [5,57].

**Figure 7:**
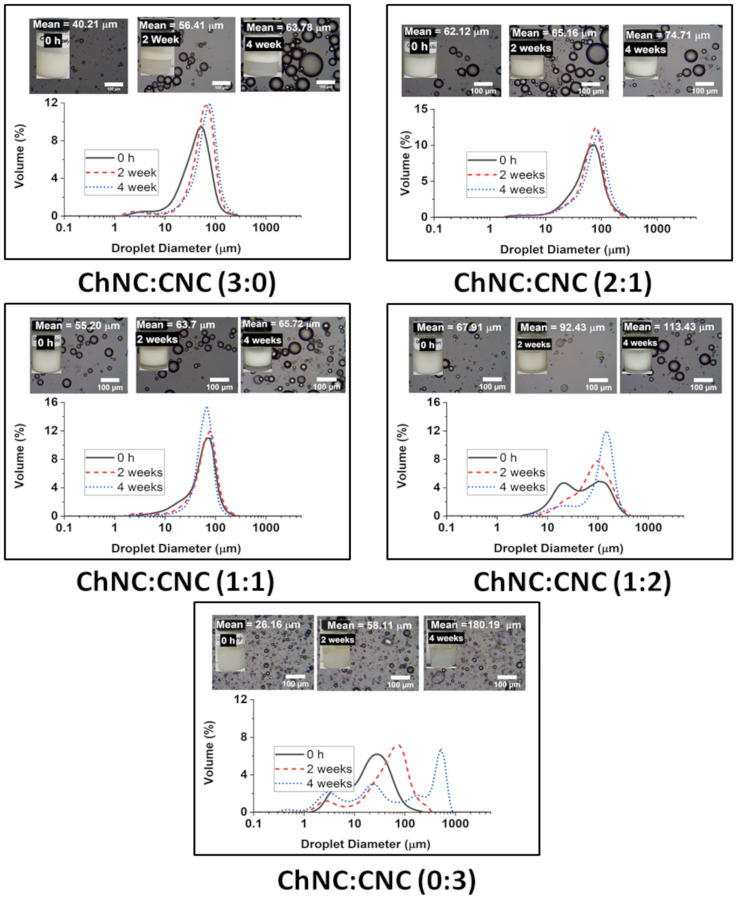
Droplet size distribution of soybean oil emulsions prepared using different ChNC:CNC mass ratios over a period of 4 weeks. The insets show optical microscopy images of each sample and pictures of the emulsions inside vials. The experiments were performed at just 20 mM NaCl, and a total NP concentration of 0.8 wt%.

A more detailed coalescence and creaming study was performed to establish the effect of ChNC:CNC mass ratio on emulsion stability (Figure 7). For this system, Ostwald ripening is not expected to play a major role, mostly due to the very low solubility that is expected for soybean oil in water and the relatively large drop diameter (low curvature). Solubilities of soybean oil in water are difficult to obtain as the oil is a complex mixture of multiple triglycerides and fatty acids. However, a few studies have reported solubilities in ethanol/water mixtures and glycerol by approximating the oil to a single average triglyceride. Even in 70% ethanol, the soluble mass fraction is just 0.001 at 25 °C, which is the same solubility in pure glycerol at 27 °C [58,59]. Our group measured the solubility of castor oil in water to be just 0.000013 ± 0.000009 g/g using HPLC; for soybean oil, it was not possible to perform the measurement; however, the solubility is expected to be even smaller as ricinoleic acid (main component of castor oil) has a secondary hydroxyl group on the 12th carbon that slightly enhances its affinity toward water.

For the emulsion stability studies, the salt concentration was maintained at just 20 mM, and the total nanoconjugate concentration was 0.8 wt%. Such a low salt concentration is adequate for food-grade emulsions, while the amount of nanocrystals is maintained as low as possible to lower the potential impact on the cost of a commercial product. After two and four weeks, the emulsions formed using CNC alone (0:3) were clearly unstable to the eye, and with significant coalescence of the remaining droplets with sizes growing from 26.16 µm (σ = 23.57 µm) to 180.19 µm (σ = 213.35 µm) over 4 weeks. Emulsions prepared using ChNC (3:0) alone were comparatively more stable to coalescence, albeit with significant creaming. In that case, the drop size increment was from 40.21 µm (σ = 28.45 µm) to 63.78 µm (σ = 34.63 µm) in the same timeframe. The highest stability was found for samples containing both CNCs and ChNCs. There was very little change in the droplet size of the emulsions prepared with ChNC-CNC nanoconjugates at 1:1, and 2:1 over a 4-week storage. The average size of their emulsion drops only grew from 55.20 µm (σ = 31.64 µm) to 65.72 µm (σ = 26.90 µm) and 62.12 µm (σ = 37.52 µm) to 74.71 µm (σ = 40.84 µm) respectively. However, the 1:2 sample did coalesce significantly, with the drops growing from 67.91 µm (σ = 61.70) to 113.43 µm (σ = 63.73), even though the emulsion appeared to remain stable after enough coalescence occurred, presumably following an arrested coalescence behavior. These results appeared to be consistent with the enhanced contact angle of the mixed samples compared to pure ChNC or CNC, as shown in Figure 6. The higher contact angle is expected to result in a higher adsorption energy [5]; unfortunately, existing methods to calculate adsorption energy from contact angle are not applicable to this complex system of two mixed-rod-shaped particles with surfaces of different wettability.

**Figure 8:**
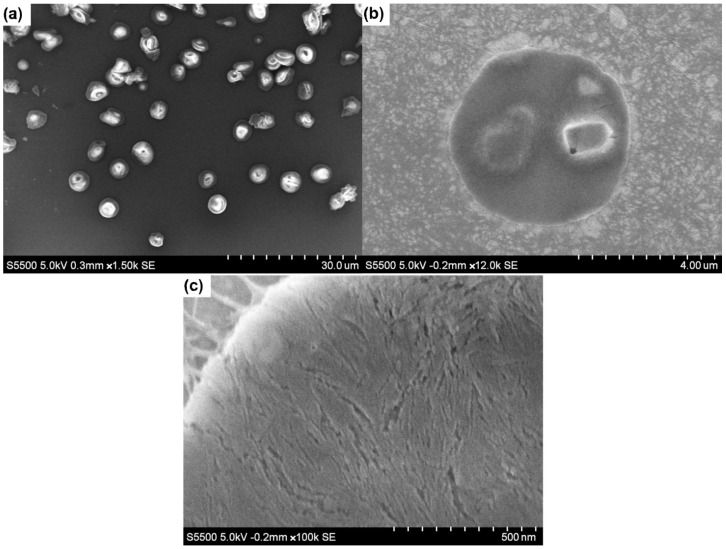
SEM images of n-heptane emulsions prepared using 1:1 ChNC-CNC nanoconjugates and dried using critical-point drying. (**a**) Multiple droplets observed; (**b**) an approximation onto a single nanoparticle crust; (**c**) a region where individual nanocrystals are oriented in a preferred direction.

The structural orientation of nanoparticles, at the surface of emulsion droplets, was studied using SEM images of 1:1 emulsion samples dried using critical-point drying (CPD), leaving behind ChNC:CNC crusts deposited on TEM grids (Figure 8). However, these were prepared with n-heptane as the oil phase, due to the extremely low volatility of the soybean oil, which prevented proper drying of the oil phase. It can be seen in Figure 8a that most emulsion droplets were structurally intact after the CPD process, while some crust broke during the drying process and others exhibited deformation. A close-up view of one crust (Figure 8c) showed the presence of regions where the nanocrystals were oriented in a preferred orientation approximately parallel to each other and tangentially to the droplet interface. This network of nanoparticles contributes to emulsions stability by restricting the movement of emulsion droplets, hence stabilizing them against droplet coalescence [16,60].

## 4. Conclusions

The morphology, interactions, and ability to stabilize emulsions of rod-like ChNC and CNC nanoconjugates (ChNC/CNC) were studied at various chitin-to-CNC ratios. Measurements of ζ-potential showed that CNCs were negatively charged in a wide range of pH (3 to 10), while ChNCs had an isoelectric point at around pH 8, below which ChNCs were positively charged. At pH 6, the ChNC:CNC conjugates with a mass ratio of 1:1 were electroneutral, resulting in aggregation. Significant aggregation was also observed for the 2:1 and 1:2 mass ratios despite ζ-potentials of 21 ± 5 mV and −33.3 ± 1 mV, respectively.

The interactions between ChNC and CNC at pH 6 were analyzed by Raman spectroscopy, providing direct evidence for the interaction between negatively charged sulfate groups on the CNC surface and the positive ammonium groups on the ChNCs. In addition, Raman signal maps of ChNC and CNC characteristic vibrations in a 1:1 mass ratio film revealed that both NPs distributed in alternated, micron-long regions, of irregular but elongated shape. Contact angle (θ) measurements on multiple ChNC/CNC films indicated a maximum θ of 80.6 ± 0.8° at a 1:1 mass ratio, providing evidence for a preferential exposure of the nanocrystals’ hydrophobic planes when both NPs were combined. Such a high contact angle suggested that the optimal mass ratio for stabilizing soybean oil–water Pickering emulsions was 1:1, which was further confirmed by emulsion stability studies.

By mixing ChNC and CNC at a 1:1 mass ratio, it was possible to produce highly stable soybean oil-in-water emulsions at a salt concentration of only 20 mM NaCl and a total NP concentration of 0.8 wt%. At these conditions, neither the CNCs nor ChNCs on their own could provide long-term stability, yet the nanoconjugates provided highly stable emulsions with emulsion drop sizes growing only 10 μm over a period of 4 weeks. The work herein presented suggest that nanoconjugates of ChNC and CNC have significant potential to provide enhanced long-term stability to food-grade emulsions in a variety of products that may include mayonnaise, salad dressings, nutritional drinks, sauces, and more.

## Data Availability

The data presented in this study are available through email upon request to the corresponding author.

## Supplementary Materials

The following supporting information can be downloaded at: https://www.mdpi.com/article/10.3390/ma15196673/s1, Figure S1. Preliminary screening of emulsion stability with varying NaCl concentration. Figure S2. Preliminary screening of emulsion stability with individual NP concentrations. Figure S3. Preliminary screening of emulsion stability with varying ChNC:CNC mass ratio. Figure S4. Long-term emulsion stability screening with varying total NP concentration. Figure S5. CNC (left) and ChNC (right) length distributions. Figure S6. Conductometric titration of (a) ChNC and (b) CNC. Figure S7. Complete Raman spectra of CNC, CNC, and ChNC-CNC mixtures from 200 to 3600 cm^−1^. Figure S8. (a) Raman spectra of ChNC, CNC, and ChNC/CNC mixtures from 200 to 1200 cm^−1^. (b) Raman spectra showing bands used for Raman mapping. Figure S9. Raman maps of a 1:1 ChNC/CNC thin film on mica.
