# Supplementary material for: Effect of the Interactions between Oppositely Charged Cellulose Nanocrystals (CNCs) and Chitin Nanocrystals (ChNCs) on the Enhanced Stability of Soybean Oil-in-Water Emulsions
Source: Materials (Basel). 2022 Sep 26;15(19):6673. doi: 10.3390/ma15196673 (PMC9573157; doi:10.3390/ma15196673)
Supplement: Supplementary file 1 [file materials-15-06673-s001.zip › materials-1896129-supplementary.pdf]

SUPPLEMENTARY MATERIALS FOR

Effect of the Interactions between Oppositely Charged Cellulose Nanocrystals (CNCs) and Chitin Nanocrystals (ChNCs) on the Enhanced Stability of Soybean Oil-in-Water Emulsions

Sanjiv Parajuli, Mohammad Jahid Hasan and Esteban E. Ureña-Benavides *

Department of Biomedical Engineering and Chemical Engineering, The University of Texas at San Antonio, San Antonio, TX 78249, USA

* Correspondence: esteban.urena-benavides@utsa.edu

Table of Contents

Preliminary Emulsion Stability Screening 4
ChNC and CNC Size Distributions 8
ChNC and CNC Conductometric Titrations 9
Raman Spectroscopy and Raman Maps 10
References 12

Table of Figures

Figure S1. Preliminary screening of emulsion stability with varying NaCl concentration from 0 to 100 mM, using 0.2 wt% ChNC in the aqeous phase. The oil/water volume ratio is 20/80. 4

Figure S2. Preliminary screening of emulsion stability with NP concentrations from 0.05 to 1.0 wt% and a NaCl concentration of 20 mM. The oil/water volume ratio is 80/20. 5

Figure S3. Preliminary screening of emulsion stability with varying ChNC:CNC mass ratio (3:0, 1:2, 1:1, 2:1 and 0:3) at 20 mM salt concentration and 0.2 wt% total NP concentration. The oil/water volume ratio is 80/20. 6

Figure S4. Long-term emulsion stability screening with varying total NP concentration (0.1% to 0.8%) at 20 mM salt concentration and ChNC/CNC mass ratio of 1:1. The oil/water volume ratio is 80/20. 7

Figure S5. CNC (left) and ChNC (right) length distributions. The insets show representative AFM topographic images for each type of NP. 8

Figure S6. Conductometric titration of a) ChNC, and b) CNC. 9

Figure S7. Complete Raman spectra of CNC, CNC and ChNC-CNC mixtures from 200 to 3600 cm^{-1}. 10

Figure S8. a) Raman spectra of ChNC, CNC and ChNC/CNC mixtures from 200 to 1200 cm^{-1}. The peaks at 262, 402 and 702 cm^{-1} correspond to the mica substrate, which is detected only in the mixed ChNC/CNC film. b) Raman spectra showing the bands used for Raman mapping, red: 230-290 cm^{-1}, blue: 1050-1150 cm^{-1}, and green: 1600-1700cm^{-1}. 10

Figure S9. Raman maps of a 1:1 ChNC/CNC thin film on mica. The colors represent Raman signals in the regions 230-290 cm^{-1} (red), 1050-1150 cm^{-1} (blue), and 1600-1700cm^{-1} (green). 11

Preliminary Emulsion Stability Screening

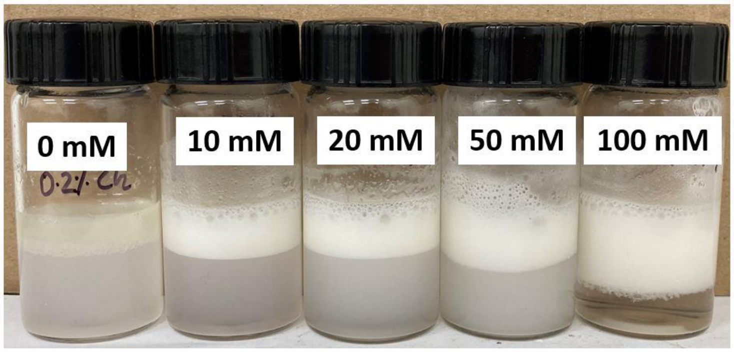

0 h

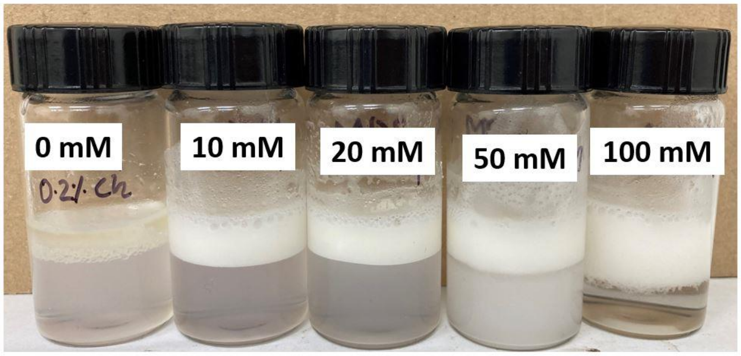

24 h

Figure S1. Preliminary screening of emulsion stability with varying NaCl concentration from 0 to 100 mM, using 0.2 wt% ChNC in the aqueous phase. The oil/water volume ratio is 20/80.

The stability of the soybean oil/water Pickering emulsions stabilized by ChNC increase with salt concentration. While samples with no added salt are highly unstable, addition of just 10 mM provides some degree of emulsifying ability; although with very significant creaming in a matter of a few minutes. Only 35% of the sample volume is emulsified. Visible turbidity in the bottom aqueous phase indicates the presence of aggregated ChNC not in the emulsion phase. At 100 mM NaCl, the clear aqueous phase at the bottom provides qualitative evidence that most ChNC are in the emulsion phase. A salt concentration of 20 mM is chosen for the following preliminary screening experiments.

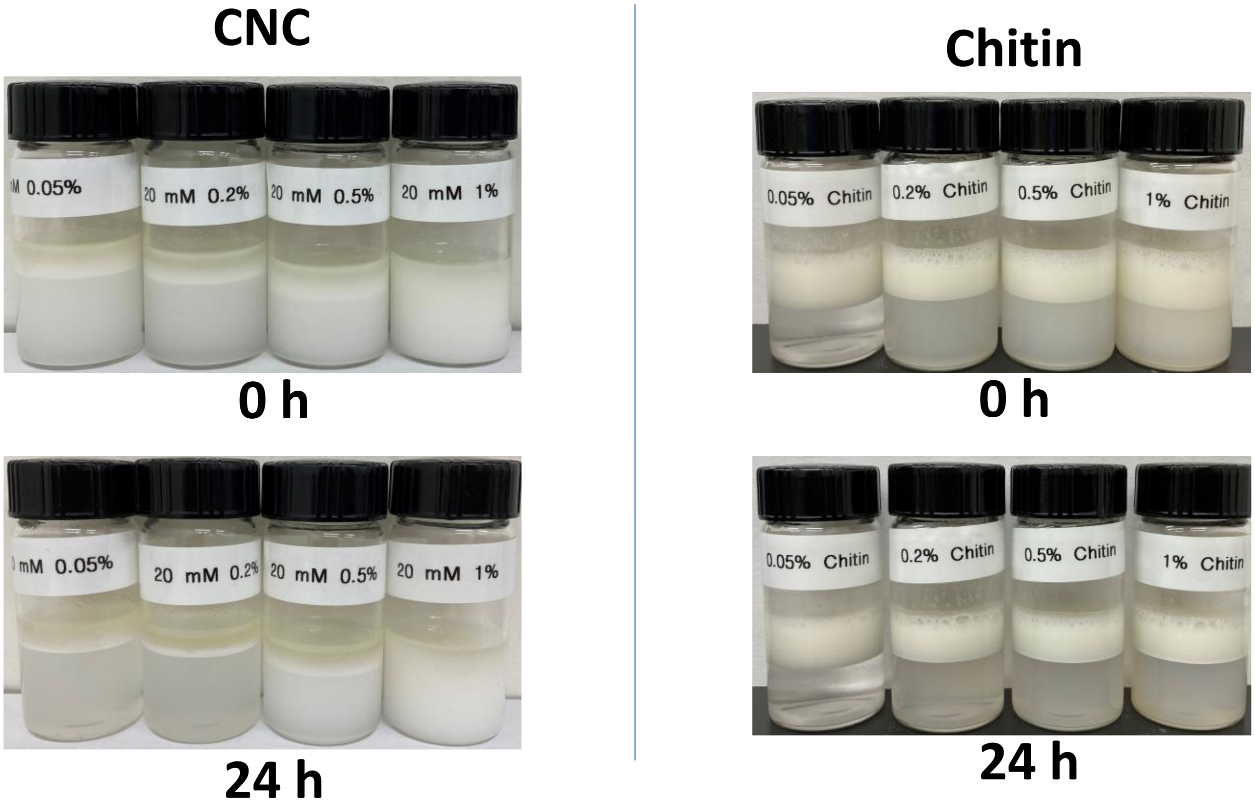

Figure S2. Preliminary screening of emulsion stability with individual NP concentrations from 0.05 to 1.0 wt% and a NaCl concentration of 20 mM. The oil/water volume ratio is 80/20.

The mixtures with CNC in Figure S2 are mostly unstable in the concentration range studied. In the case of ChNC, the emulsifying ability is significantly better. Interestingly there is no appreciable effect on creaming as the NP concentration is varied from 0.05 to 1.0 wt%; the volume of the emulsion phase after 24 h is 39% of the total mixture in all cases. A low NP concentration of 0.2 wt% is chosen for screening the ChNC:CNC mass ratio.

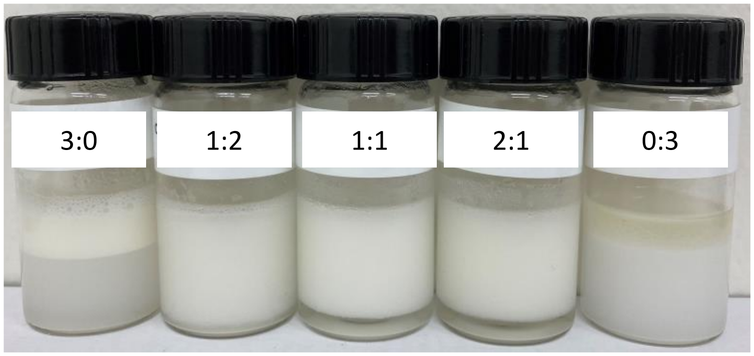

0 h

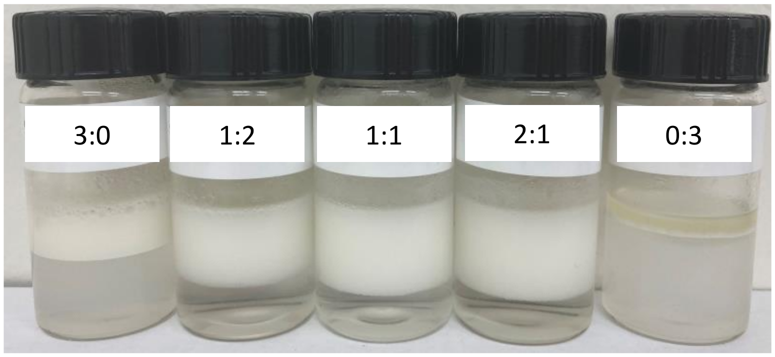

24 h

Figure S3. Preliminary screening of emulsion stability with varying ChNC:CNC mass ratio (3:0, 1:2, 1:1, 2:1 and 0:3) at 20 mM salt concentration and 0.2 wt% total NP concentration. The oil/water volume ratio is 80/20.

The preliminary screening of ChNC:CNC mass ratio indicates that the mixed samples (1:2, 1:1, and 2:1) all have significantly higher stability based on creaming compared to using CNC or ChNC only. However, the amount of creaming after 24 h is still higher than desired, with the emulsion phase comprising only 65% of the total sample volume. A mas ratio of 1:1 is chosen for an additional long-term screening of NP concentration, which is required to find a concentration providing adequate stability to creaming.

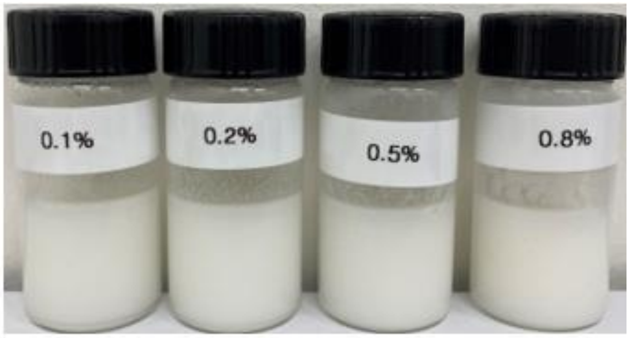

0 h

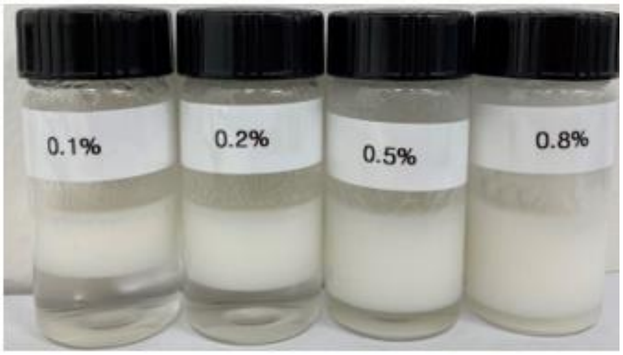

2 Weeks

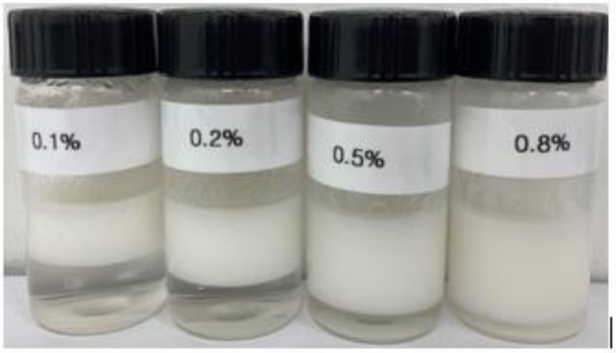

4 Weeks

Figure S4. Long-term emulsion stability screening with varying total NP concentration (0.1% to 0.8%) at 20 mM salt concentration and ChNC/CNC mass ratio of 1:1. The oil/water volume ratio is 80/20.

In the case of the mixed ChNC/CNC samples, the stability to creaming over time is significantly impacted by the NP concentration as shown in Figure S4. The emulsion phase volumes after 4 weeks are 47%, 59%, 76%, and 86% of the total sample for NP concentrations of 0.1, 0.2, 0.5, and 0.8 wt%, respectively. The 86% emulsification after 4 weeks is appropriate for this study, and thus a NP concentration of 0.8 wt % is chosen for the more rigorous emulsion stability studies.

ChNC and CNC Size Distributions

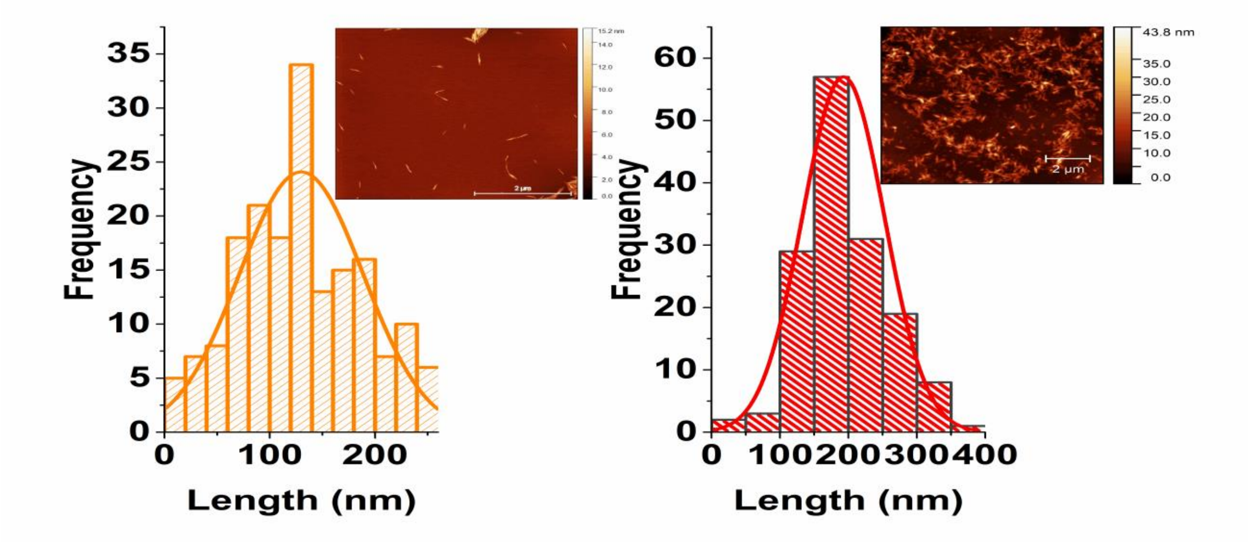

Figure S5. CNC (left) and ChNC (right) length distributions. The insets show representative AFM topographic images for each type of NP.

The length of at least 175 nanocrystals was measured using atomic force microscopy images. The widths were approximately 6 to 8 nm for both types of nanocrystals; however these values are less accurate than the lengths, due to the impossibility of completely eliminating tip broadening effects.

ChNC and CNC Conductometric Titrations

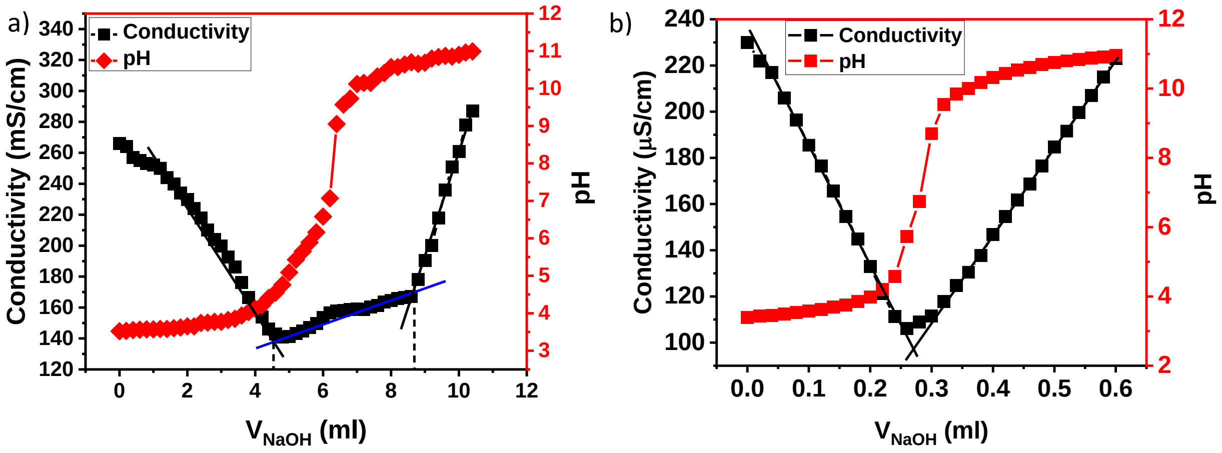

Figure S6. Conductometric titration of a) ChNC, and b) CNC.

Titration Methods

In the case of ChNC, a dispersion of 1.71 wt% was prepared in a 0.01 M HCl solution, which resulted in an initial pH of 2.9. The sample was then adjusted to a pH of 3.5 using NaOH. The titration was done with continuous mixing by adding 0.2 ml increments of 0.02 M NaOH, while tracking the pH and conductivity of the aqueous dispersion. The initial downward slope of the conductivity trace corresponds to neutralization of excess acid, the intermediate region represents the neutralization of ammonium groups on the ChNC surface, while the last section is caused by an increased conductivity from the addition of Na^+ and -OH ions to the mixture[1]. The surface charge and degree of deacetylation is calculated based on the base moles consumed in the intermediate region. In the case of CNC, the transition between excess acid titration and sulfate group titration is mostly undetectable. Thus, CNCs were first acidified by dialyzing against 1 M HCl to protonate all sulfate groups. The sample was then dialyzed against DI water until constant pH to remove excess acid. A 0.2 wt% CNC sample was then prepared in deionized water and titrated against 0. 05 M NaOH using 0.02 ml increments.

Titration Results

The titration yielded a ChNC degree of deacetylation of 13.0% (charge: 4.11×10^{20} e-/g ChNC); while the CNC had a sulfur content of 0.435% (charge: 8.45×10^{19} e-/g ChNC).

Raman Spectroscopy and Raman Maps

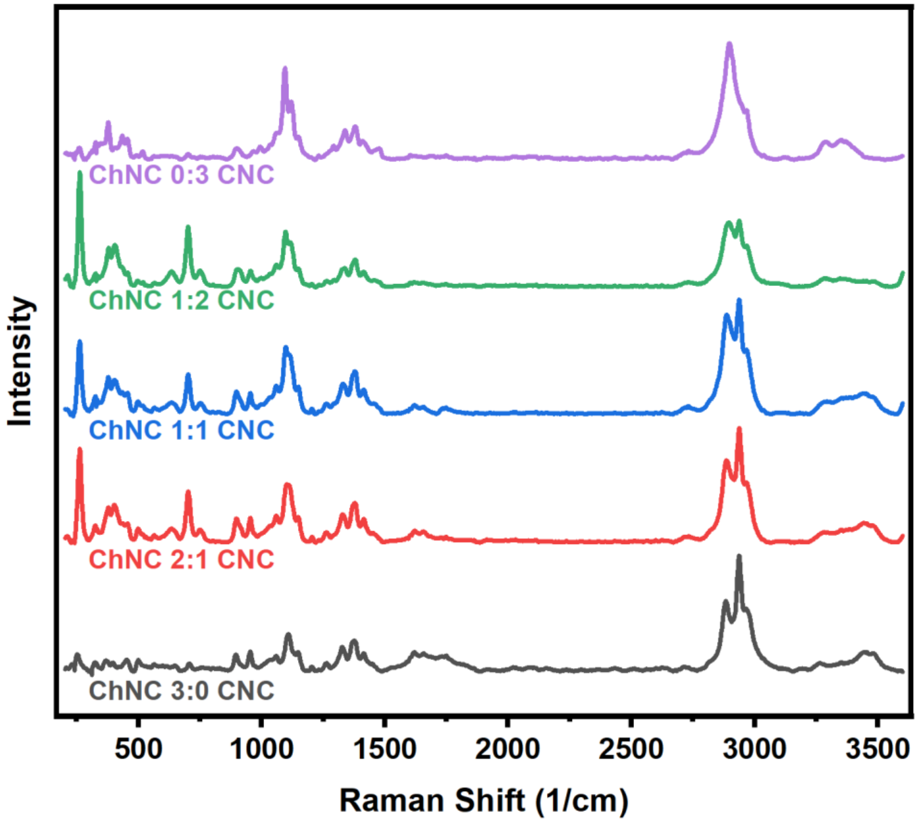

Figure S7. Complete Raman spectra of CNC, CNC and ChNC-CNC mixtures from 200 to 3600 cm^{-1}.

a)

b)

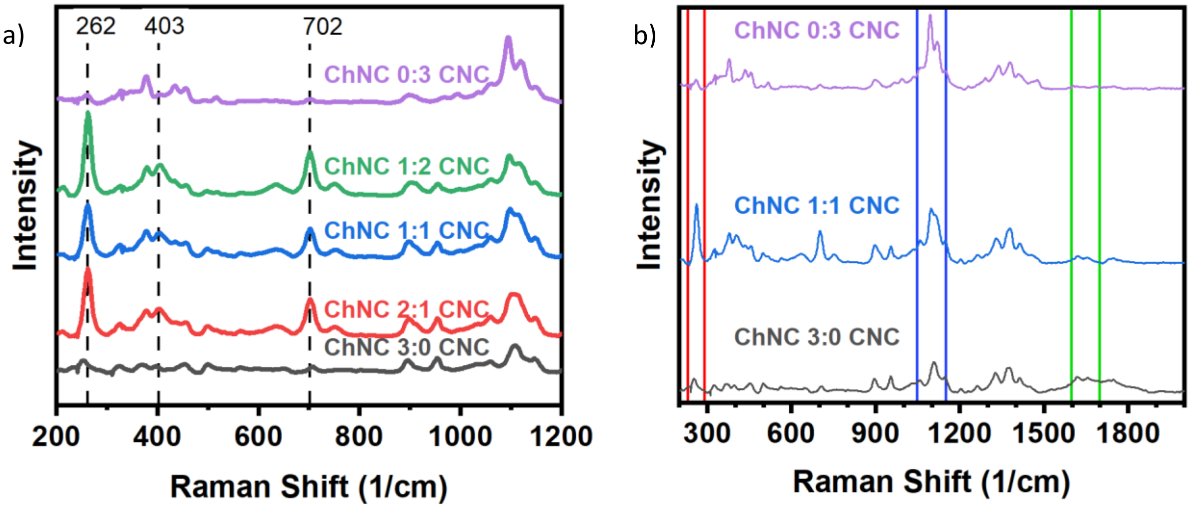

Figure S8. a) Raman spectra of ChNC, CNC and ChNC/CNC mixtures from 200 to 1200 cm^{-1}. The peaks at 262, 402 and 702 cm^{-1} correspond to the mica substrate, which is detected only in the mixed ChNC/CNC film. b) Raman spectra showing the bands used for Raman mapping, red: 230-290 cm^{-1}, blue: 1050-1150 cm^{-1}, and green: 1600-1700cm^{-1}.

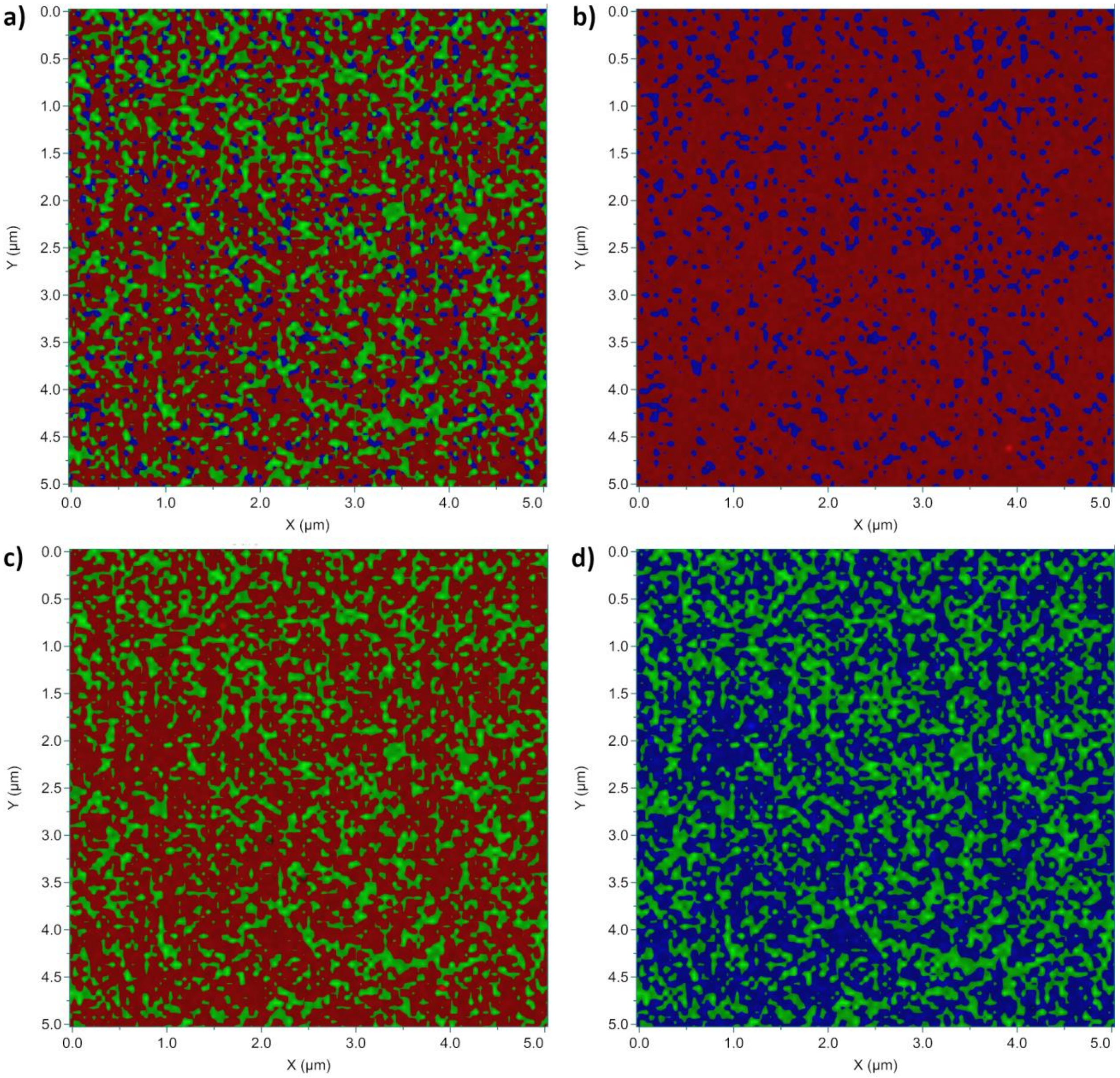

Figure S9. Raman maps of a 1:1 ChNC/CNC thin film on mica. The colors represent Raman signals in the regions 230-290 cm^{-1} (red), 1050-1150 cm^{-1} (blue), and 1600-1700cm^{-1} (green).

Full Raman spectra of CNC, ChNC and ChNC/CNC films is shown in Figure S7, while narrower range spectra are observed in Figure S8. The typical Raman bands of the mica substrate are observed in Figure S8a; these show up at 262, 403, and 702 cm^{-1}. In order to investigate the degree of coverage of the mica sheet by the nanocrystals and their distribution over the surface, a Raman signal map was created (Figure S9). The color codes of the Raman maps are described in Figure S8b over representative Raman spectra of pure CNC, 1:1 ChNC/CNC, and pure ChNC.

The red regions (230-290 cm^{-1}) represent the detection of mica, green (1600-1700cm^{-1}) arises from the carbonyl vibration bands in ChNC, while the blue (1050-1150 cm^{-1}) regions correspond to skeletal

bending and stretching, as well as glycosidic O-C-O bond vibration [2]. The blue signal is much stronger in CNCs, however it is also present with lower intensity in ChNC, as seen in Figure S8b, thus it can be interpreted as a region containing either CNC or ChNC. Nevertheless, the green signal is distinct of ChNC.

In the blue/green map of Figure S9d, the blue can be interpreted as regions with CNC, while the green corresponds to ChNC. The 5.0x5.0 μm map show the presence of irregularly shaped, submicron sized, green regions arising from moderately aggregated ChNCs, intercalated with regions of CNCs of similar size. Comparison of Figures S9a, S9c, and S9d reveal the green regions in all maps have the same size, shape and position. The maps in Figures S9a and S9c show red regions arising from mica in mostly the same sections where blue appears in Figure S9d; moreover, Figure S9b is mostly red with just a few small pockets of blue. This is possibly due to the mica signal being so strong that it overpowers the less intense blue range vibrations. The blue regions in Figure S9b are presumably sections where the film is thicker, such that the laser intensity reaching the mica substrate is not high enough to generate a significant mica signal.

References

(1) dos Santos, Z. M.; Caroni, A. L. P. F.; Pereira, M. R.; da Silva, D. R.; Fonseca, J. L. C. Determination of Deacetylation Degree of Chitosan: A Comparison between Conductometric Titration and CHN Elemental Analysis. *Carbohydrate Research* **2009**, *344* (18), 2591–2595. https://doi.org/10.1016/j.carres.2009.08.030.

(2) Makarem, M.; Lee, C. M.; Kafle, K.; Huang, S.; Chae, I.; Yang, H.; Kubicki, J. D.; Kim, S. H. Probing Cellulose Structures with Vibrational Spectroscopy. *Cellulose* **2019**, *26* (1), 35–79. https://doi.org/10.1007/s10570-018-2199-z.
